# Upregulation of MicroRNA-19b predicts good prognosis in patients with hepatocellular carcinoma presenting with vascular invasion or multifocal disease

**DOI:** 10.1186/s12885-015-1671-5

**Published:** 2015-10-09

**Authors:** Chung-Lin Hung, Chia-Shen Yen, Hung-Wen Tsai, Yu-Chieh Su, Chia-Jui Yen

**Affiliations:** 1 Division of Oncology and Hematology, Department of Internal Medicine, Buddhist Dalin Tzu Chi General Hospital, Chiayi, 600 Taiwan; 2 Division of General Surgery, Department of Surgery, Chi Mei Medical Center, Tainan, 704 Taiwan; 3 Department of Pathology, National Cheng Kung University Hospital, College of Medicine, National Cheng Kung University, Tainan, 704 Taiwan; 4 Division of Hematology-Oncology, Buddhist Dalin Tzu Chi Hospital, Taiwan, ROC; 5 School of Medicine, Tzu Chi University, Hualien, Taiwan, ROC; 6 Division of Hematology and Oncology, Department of Internal Medicine, National Cheng Kung University Hospital, College of Medicine, National Cheng Kung University, 138 Sheng-Li Road, Tainan, 704 Taiwan

**Keywords:** Multifocal, Vascular invasion, miR-19b, MAPK14, HIF1A

## Abstract

**Background:**

After surgical resection of hepatocellular carcinoma (HCC), recurrence is common, especially in patients presenting with vascular invasion or multifocal disease after curative surgery. Consequently, we examined the expression pattern and prognostic value of miR-19b in samples from these patients.

**Methods:**

We performed a miRNA microarray to detect differential expression of microRNAs (miRNAs) in 5 paired samples of HCC and non-tumoral adjacent liver tissue and a quantitative real-time polymerase chain reaction (PCR) analysis to validate the results in 81 paired samples of HCC and adjacent non-tumoral liver tissues. We examined the associations of miR-19b expression with clinicopathological parameters and survival. MiR-19b was knocked down in Hep3B and an mRNA microarray was performed to detect the affected genes.

**Results:**

In both the miRNA microarray and real-time PCR, miR-19b was significantly overexpressed in the HCC tumor compared with adjacent non-tumor liver tissues (*P* < 0.001). The expression of miR-19b was significantly higher in patients who were disease-free 2 years after surgery (*P* < 0.001). High miR-19b expression levels were associated with higher α-fetoprotein levels (*P* = 0.017). In the log-rank test, high miR-19b was associated with better disease-free survival (median survival 37.107 vs. 11.357; *P* = 0.022). In Cox multivariate analysis, high miR-19b predicted better disease-free survival and overall survival (hazards ratio [HR] = 0.453, 95 % confidence interval [CI] = 0.245–0.845, *P* = 0.013; HR = 0.318, CI = 0.120–0.846, *P* = 0.022, respectively). N-myc downstream regulated 1 (NDRG1) was downregulated, while epithelial cell adhesion molecule (EPCAM), hypoxia-inducible factor 1-alpha (HIF1A), high-mobility group protein B2 (HMGB2), and mitogen activated protein kinase 14 (MAPK14) were upregulated when miR-19b was knocked down in Hep3B.

**Conclusions:**

The overexpression of miR-19b was significantly correlated with better disease-free and overall survival in patients with HCC presenting with vascular invasion or multifocal disease after curative surgery. MiR-19b may influence the expression of NDRG1, EPCAM, HMGB2, HIF1A, and MAPK14.

**Electronic supplementary material:**

The online version of this article (doi:10.1186/s12885-015-1671-5) contains supplementary material, which is available to authorized users.

## Background

Hepatocellular carcinoma (HCC) is the sixth most prevalent cancer worldwide, and the third most common cause of cancer-related deaths [1]. In eastern Asian countries, including Taiwan, chronic infection with hepatitis B virus (HBV) is the dominant risk factor [2, 3]. Among treatment options, surgical resection of the tumor remains one of the most effective ways to cure HCC. Traditionally, patients with clinical Barcelona-Clinic Liver Cancer (BCLC) stage A disease are candidates for surgery. However, several reports have shown that curative surgery provides benefits even in patients with vascular invasion or multifocal diseases [4, 5]. Recurrence remains the main cause of treatment failure, with recurrence rates up to 70 % within 5 years after surgery [6]. Risk stratification of patients receiving surgery and identification of high-risk groups are major challenges. Prognostic factors focusing on this group of patients have been limited.

MicroRNAs (miRNAs) are small, non-coding RNAs composed of ~21 nucleotides. They are transcribed as precursors in the nucleus and are subsequently processed into mature miRNAs in the cytoplasm. Mature miRNAs bind to the 3′-untranslated region of target messenger RNAs (mRNAs), resulting in translational suppression or degradation of the mRNAs [7]. The role of miRNAs in cancer has often been discussed. Several miRNAs, including miR-21, are known to be oncogenic, while the let-7 family has been revealed as a tumor suppressor [8, 9]. A growing amount of evidence has suggested that miRNAs play important roles as prognostic and predictive biomarkers in cancers. MiR-21-5p, miR-20a-5p, miR-103a-3p, miR-106b-5p, miR-143-5p, and miR-215 could stratify risk groups among stage II colon cancer patients [10]. MiR-1290, miR-196b, and miR-135a* have been shown to predict the chemotherapy response patients with lung adenocarcinoma [11]. Several miRNAs have also been reported to correlate with the disease severity and prognosis of HCC, including miR-15b, miR-122 and miR-29 [12–14].

MiR-19b is a member of the miR-17-92 cluster. In the literature, miR-19b has been shown to play a role in the aging process and thrombosis, as well as cardiovascular diseases [15–18], and is deregulated in several cancers, including breast cancer, lung cancer, glioma, and cervical cancer [19–22]. Some reports have suggested that miR-19b is upregulated in cancer cells and promotes proliferation and chemoresistance, while others revealed its ability to suppress angiogenesis and migration [23–25]. The role of miR-19b in HCC has not been elucidated.

In the present study, we investigated the feasibility of miR-19b as a novel prognostic factor for hepatitis B virus (HBV)-associated HCC with multifocal disease or vascular invasion after curative surgery.

## Methods

### Patients and tissue samples

We retrospectively investigated 81 patients diagnosed with HCC and HBV who had either BCLC stage B or stage C disease without extrahepatic metastases who received curative surgery between June 2007 and October 2013 at National Cheng Kung University Hospital. For each case, the diagnosis, histologic grade, and presence of liver cirrhosis were confirmed by pathologists. HBV infection was diagnosed by the presence of serum HBV surface antigen. None of these patients had received chemotherapy or radiotherapy before surgery. Snap-fresh HCC tissues and paired adjacent non-tumorous liver tissues were obtained from each patient during surgery. Tissues were stored in liquid nitrogen after surgical resection until use. HCC tissues were collected from surgical resected samples presenting with tumorous features macroscopically. Adjacent non-tumor tissues were collected > 2 cm away from the edge of the tumors. Clinical parameters including the serum α-fetoprotein (AFP) level at diagnosis, age, TNM stage, and gender were obtained from the database of National Cheng Kung University Hospital Cancer Center. An abdominal computed tomography scan or magnetic resonance imaging was performed every 3 to 4 months after surgery to detect recurrence. The present study was approved by the Institutional Review Board of National Cheng Kung University Hospital (ER-99-251). Written informed consent was obtained from all patients. All specimens were handled anonymously according to legal and ethical regulations, and in accordance with the Helsinki Declaration of 1975, as revised in 1983. The clinicopathological features of the patients are summarized in Table 1 and Additional file 1: Table S1.

**Table 1:** Correlation of miR-19b expression with clinicopathological features of hepatocellular carcinoma

		miR-19b expression		
Clinicopathological variables	Number of cases	High	Low	*P*
Age				
> 60	33(40.7)	16(40)	17(41.5)	0.893
< 60	48(59.3)	24(60)	24(58.5)	
Gender				
Male	55(67.9)	25(62.5)	30(73.2)	0.304
Female	26(32.1)	15(37.5)	11(26.8)	
TNM stage				
II	58(71.6)	31(77.5)	27(65.9)	0.245
III + IV	23(28.4)	9(22.5)	14(34.1)	
Liver cirrhosis				
Yes	26(32.1)	15(37.5)	11(26.8)	0.304
No	55(67.9)	25(62.5)	30(73.2)	
Vascular invasion^a^				
Presence	62(76.5)	29(72.5)	33(80.5)	0.369
Absence	19(23.5)	11(27.5)	8(19.5)	
AFP (ng/ml)				
> 20	48(59.3)	29(72.5)	19(46.3)	0.017
< 20	33(40.7)	11(27.5)	22(53.7)	
Tumor differentiation				
W + M	66(81.5)	32(80)	34(82.9)	0.735
P	15(18.5)	8(20)	7(17.1)	
Tumor number				
Solitary	58(71.6)	30(75)	28(68.3)	0.503
Multiple	23(28.4)	10(25)	13(31.7)	

*AFP*, α-fetoprotein, *W* well differentiated, *M* moderate differentiated, *P* Poorly differentiated, *BCLC* Barcelona Clinic Liver Cancer

^a^Presence of vascular invasion represented BCLC stage C; absence of vascular invasion represented BCLC stage B

### Isolation of total RNA

Total RNA was isolated from frozen samples using miRNA isolation kits (Qiagen®, Germantown, MD, USA) according to the manufacturer’s protocol. Briefly, around 30 mg of snap-fresh tissue of HCC or adjacent non-tumorous liver were disrupted and homogenized. The lysate was then centrifuged and the supernatant was transferred to the gDNA Eliminator spin column. After centrifugation, the flow-through was transferred to the RNeasy spin column. RNA was extracted using the buffers RPE and RW1. The gDNA Eliminator spin columns, RNeasy spin column and buffers were all supplied in the Qiagen miRNA isolation kits. The concentration and quality of total RNA were measured by NanoDrop ND-1000 (NanoDrop Technologies, Wilmington, DE, USA) at 260 and 280 nm (A260/280) and confirmed by gel electrophoresis.

### Human sample microRNA microarray

We selected 5 patients with HBV-associated HCC and performed a miRNA microarray. Two of these patients had liver cirrhosis. RNA labeling and hybridization were completed using a kit from Welgene Biotech Co., Ltd (Welgene Biotech Co., Ltd., Taipei, Taiwan, R.O.C) according to the manufacturer’s instructions. Briefly, RNA was extracted using miRNA isolation kits (Qiagen®) according to the manufacturer’s protocol. RNA purified was quantified at OD 260 nm by an ND-1000 spectrophotometer (NanoDrop Technologies) and analyzed by the Bioanalyzer 2100 (Agilent Technologies, Santa Clara, CA, USA) with the RNA 6000 Nano LabChip kit. During the in vitro transcription process, 1 μg of total RNA was amplified by a low RNA input fluor linear amp kit (Agilent) and labeled with Cy3 (CyDye, PerkinElmer, Waltham, MA, USA). Using incubation with fragmentation buffer at 60 °C for 30 min, 1.65 μg of Cy3-labled cRNA was fragmented to an average size of about 50–100 nucleotides. Correspondingly fragmented labeled cRNA was then pooled and hybridized to SurePrint G3 ChIP/CH3 1X1M array (Agilent) at 60 °C for 17 h. After washing and drying by nitrogen gun blowing, the microarrays were scanned with an Agilent microarray scanner at 535 nm for Cy3. Scanned images were analyzed by Agilent Feature Extraction, version 10.5. Image analysis and normalization software were used to quantify the signal and background intensity for each feature. The data have been deposited in NCBI’s Gene Expression Omnibus and are accessible through GEO Series accession no. GSE69580.

### Cell line mRNA microarray

RNA labeling and hybridization were completed using a kit from Phalanx Biotech Co., Ltd. (Phalanx Biotech Group, Inc., Hsinchu City, Taiwan, R.O.C) according to the manufacturer’s instructions. Briefly, RNA was extracted after miR-19b knockdown in Hep3B. Purified RNA was labeled with fluorescein and hybridized on Human OneArray® (Phalanx Biotech) with 29187 mature human mRNA probes. Finally, hybridization signals were detected, and the images were scanned and quantified. The data have been deposited in NCBI’s Gene Expression Omnibus and are accessible through GEO Series accession number GSE69519.

### Real time qRT-PCR analysis for miRNA expression

Complementary DNA was synthetized from the total RNA using gene-specific primers of the TaqMan MicroRNA Reverse Transcription Kit (Applied Biosystems®, Foster City, CA). For real time quantitative reverse transcription polymerase chain reaction (qRT-PCR), primers for miR-19b and endogenous control U6 were purchased from Applied Biosystems. All reactions were carried out in triplicate according to the manufacturer’s protocol. Briefly, we used 10 ng of RNA sample, 50 nmol/l of stem-loop reverse transcriptase (RT) primer, 10X RT buffer, 0.25 mmol/l each of deoxynucleotide triphosphates (dNTPs), 3.33 U/μl MultiScribe RT, and 0.25 U/μl RNase inhibitor (all from Applied Biosystems’ TaqMan MicroRNA Reverse Transcription Kit®). Reaction mixtures (15 μl) were incubated for 30 min at 16 °C, 30 min at 42 °C, and 5 min at 85 °C and then held at 4 °C (2720 Thermal Cycler; Applied Biosystems®). Real-time PCR was performed using the StepOne™ Plus Real-Time PCR System (Applied Biosystems®). The 20 μl PCR reaction mixture included 1.33 μl of RT product, 1X TaqMan Universal PCR Master Mix, and 1 μl of primer and probe mix from the TaqMan MicroRNA Assay Kit (Applied Biosystems®). Reactions were incubated in a 96-well optical plate at 95 °C for 10 min, followed by 40 cycles at 95 °C for 15 s and 60 °C for 60 s. Relative quantification of the miR-19b expression was evaluated using the comparative cycle threshold method. The raw data were presented as the relative quantity of miR-19b, normalized with respect to U6.

### Real time qRT-PCR analysis for mRNA expression

Complementary DNA was synthetized from the total RNA using gene-specific primers of the TaqMan® Reverse Transcription Kit (Applied Biosystems®, Foster City, CA, USA). For real time qRT-PCR, primers for N-myc downstream regulated 1 (NDRG1), epithelial cell adhesion molecule (EPCAM), hypoxia-inducible factor 1-alpha (HIF1A), high-mobility group protein B2 (HMGB2) and mitogen activated protein kinase 14 (MAPK14) and endogenous control glyceraldehyde 3-phosphate dehydrogenase (GAPDH) were purchased from Applied Biosystems. All reactions were carried out in triplicate according to the manufacturer’s protocol. Briefly, we used 1 ng of RNA sample, 1 μl random primer (random hexamer at a concentration of 0.5 μM as primer, 10X RT buffer, 2.5 mM each of dNTPs, 1 μl of MultiScribe RT™ at a concentration of 50 U/μl, 1.4 μl of 25 mM MgCl_2_ and 1 μl of RNase inhibitor at a concentration of 20 U/μl (all from Applied Biosystems’ TaqMan® Reverse Transcription Kit). Reaction mixtures (20 μl) were incubated for 10 min at 25 °C, 30 min at 37 °C, and 5 min at 95 °C and then held at 4 °C (2720 Thermal Cycler; Applied Biosystems®). Real-time PCR was performed using the StepOne™ Plus Real-Time PCR System (Applied Biosystems®). The 10 μl PCR reaction mixture included 1 μl of RT product, 5 μl of 2X TaqMan Universal PCR Master Mix, and 0.5 μl of primer and probe mix from the TaqMan Gene expression Assay Kit (Applied Biosystems®). Reactions were incubated in a 96-well optical plate at 95 °C for 10 min, followed by 40 cycles at 95 °C for 15 s and 60 °C for 60 s. Relative quantification of the miR-19b expression was evaluated using the comparative cycle threshold method. The raw data were presented as the relative quantity of NDRG1, EPCAM, HIF1A, HMGB2 and MAPK14, normalized with respect to GAPDH.

### Cell line culture

Human HCC cell line Hep 3B was obtained from American Type Culture Collection (ATCC®, Manassas, VA, USA), was validated in 2014, and was cultured in MEM medium (Invitrogen, Carlsbad, CA,USA) plus 10 % newborn calf serum. Ethics approval was not required.

### Transfection

A quantity of approximately 2 × 10^5^ Hep 3B cells were seeded and cultured in 6-well plates. For each well, 90 pmol of miR-19b inhibitor or control were added to 300 μL Opti-MEM medium and 10 μL of Lipofectamine® 2000 (all Applied Biosystems®). The mixture was added to the cells and incubated for 6 h before replacing the medium. Cells were collected for RNA extraction 24 h after transfection.

### Statistical analysis

The Mann–Whitney test was performed to determine the significance of miRNA levels between the HCC tumor and non-tumor adjacent tissues. Student’s *t*-test was performed to determine the significance of the AFP level between different groups of patients. Group comparisons of categorical variables were evaluated using the *χ*^2^ test. Overall survival (OS) was defined from the date of diagnosis to the date of death. Correlation of variables was analyzed using Pearson correlation coefficient. Disease free survival (DFS) was defined from the date of surgery to the date of recurrence. Survival curves were plotted using the Kaplan–Meier method and differences in survival rates were analyzed using the log-rank test. The prognostic relevance of each variable to OS and DFS were analyzed using the Cox regression model. Multivariate analysis of the prognostic factors was performed using the Cox regression model. A *P*-value less than 0.05 was considered statistically significant. All statistical calculations were performed using SPSS 18.0 for Windows (SPSS Inc., Chicago, IL, USA).

## Results

### Overexpression of miR-19b in hepatocellular carcinoma

**Fig. 1:**
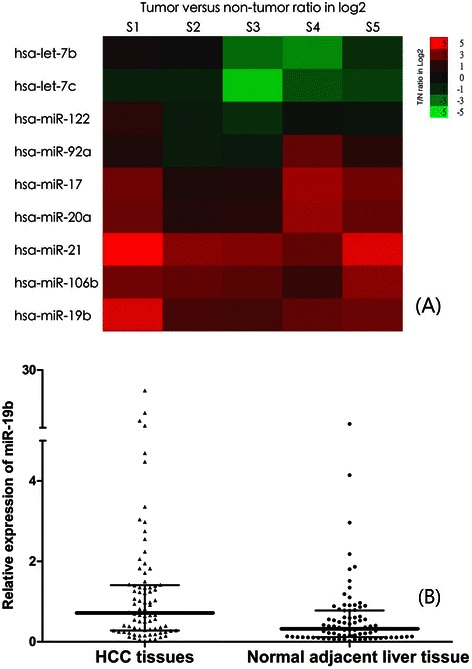
**a** MiRNAs are deregulated in hepatocellular carcinoma as detected by miRNA microarray. Five pairs of hepatocellular carcinoma and adjacent non-tumor liver tissue matches were analyzed using SurePrint G3 ChIP/CH3 1X1M array (Agilent Technologies, Santa Clara, CA, USA). Rows: miRNAs; columns: cases. For each miRNA, red represents higher expression and green represents lower expression than the corresponding adjacent non-tumoral liver tissue expression. S1, sample 1; S2, sample 2; S3, sample 3; S4, sample 4; S5, sample 5. **b** MiR-19b is overexpressed in the HCC tissues compared with normal adjacent liver tissue (median expression level 0.3246, range 0.0169 to 6.667, *P* < 0.001, Mann–Whitney test). miR-19b, microRNA-19b. HCC, hepatocellular carcinoma

**Fig. 2:**
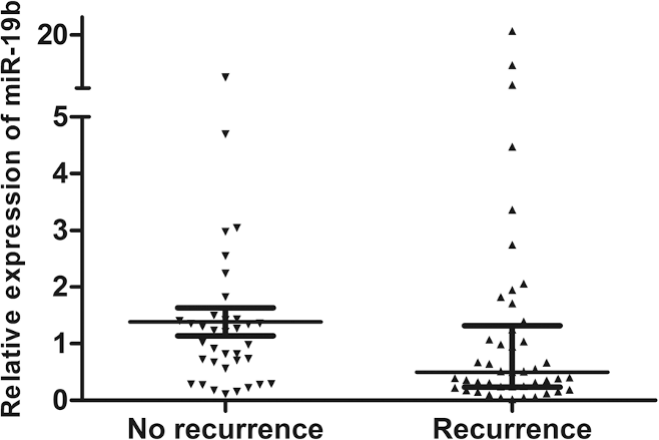
MiR-19b in HCC is overexpressed in patients with no recurrence (*n* = 36, median expression level 1.1109, range 0.0192 to 21.104) compared with those whose cancer recurred within 2 years (*n* = 45, median 0.4988, range 0.1123 to 7.997), *P* < 0.001, Mann–Whitney test. miR-19b, microRNA-19b. HCC, hepatocellular carcinoma

We first performed a miRNA microarray for 5 selected paired HCC and adjacent non-tumoral liver tissue samples. As shown in Fig. 1a and Additional file 1: Table S2, we found that miR-19b was up-regulated in all five samples. MiR-21, miR-17, miR-20a, and miR-106b were also overexpressed, whereas let-7b and let-7c were downregulated in HCC tumor samples. To validate these results, we then evaluated miR-19b expression by qRT-PCR analysis in 81 paired samples of tumor and adjacent non-tumorous tissues diagnosed with HCC. The results are shown in Fig. 1b. The expression levels of miR-19b in the HCC tumorous tissues (median expression level 0.7184, range 0.0192 to 21.104) were significantly higher than those in the adjacent non-tumorous liver tissues (median expression level 0.3246, range 0.0169 to 6.667, *P* < 0.001). We also found that the expression level of miR-19b was significantly higher in patients who were disease-free for at least two years after surgery (*n* = 36, median expression level 1.1109, range 0.0192 to 21.104), compared with those whose cancer recurred within two years (*n* = 45, median 0.4988, range 0.1123 to 7.997, *P* < 0.001, Fig. 2.).

### Association of miR-19b with the clinicopathological features of HCC

The median expression value of miR-19b was used as a cut-off. HCC tissue samples expressing miR-19b at levels lower than the median expression level were assigned to the low-expression group (*n* = 41) and samples with expression above the median value were assigned to the high-expression group (*n* = 40). The relationships of miR-19b with various clinicopathological features of HCC were analyzed and are summarized in Table 1. The results revealed that a high level of miR-19b expression was correlated with an elevated AFP level (*P* = 0.017). However, there were no significant correlations of miR-19b expression with other clinical features such as gender, age, vascular invasion, TNM stage, liver cirrhosis, tumor differentiation or number of tumors (all *P* > 0.05). There was no significant difference in the serum AFP levels between the miR-19b low-expression and high-expression groups (*Student’s t*-test, *P* = 0.408). There was no correlation between the miR-19b expression level and serum AFP level (*Pearson* correlation coefficient, r = −0.032, *p* = 0.778).

### Mir-19b expression predicts better survival in patients with HCC

**Fig. 3:**
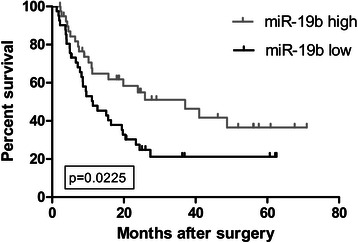
Correlation between miR-19b expression and disease-free survival rates in 81 patients with HCC after curative surgery. Patients with high levels of miR-19b had significantly better disease-free survival than those with low levels (median survival 37.107 vs.11.357; *P* = 0.022, Log-rank test. Kaplan–Meier survival curves for disease-free survival are plotted according to miR-19b expression. miR-19b, microRNA-19b. HCC, hepatocellular carcinoma

We further investigated the correlation between the miR-19b expression level and the survival of patients with HBV-associated HCC. As shown in Fig. 3, the DFS of the high miR-19b expression group was significantly longer than that of the low miR-19b expression group (median survival 37.107 vs.11.357; *P* = 0.022). In multivariate analysis, miR-19b expression was an independent good prognostic factor for both DFS (hazards ratio [HR] = 0.453, 95 % confidence interval [CI] = 0.245–0.845, *P* = 0.013, Table 2) and OS (HR = 0.318, CI =0.120–0.846, *P* = 0.022, Table 2) in patients with more advanced HBV-associated HCC.

**Table 2:** Multivariate analysis of the associations of disease-free survival and overall survival with various clinicopathologic parameters and miR-19b expression in patients with HCC presented with vascular invasion or multifocal diseases

	Disease-free survival			Overall survival		
Clinicopathological parameters	HR	95 % CI	*P* value	HR	95 % CI	*P* value
Age	0.947	0.517–1.737	0.861	0.527	0.204–1.357	0.184
Gender	0.857	0.440–1.672	0.568	2.184	0.726–6.569	0.165
AFP level	2.133	1.155–4.616	0.016	1.823	0.705–4.714	0.215
Tumor number	1.213	0.626–2.349	0.567	1.099	0.401–3.105	0.855
Vascular invasion	0.82	0.414–1.623	0.568	2.963	0.672–13.060	0.151
TNM stage	2.364	1.211–4.616	0.012	3.084	1.201–7.92	0.019
Tumor differentiation	1.481	0.647–3.258	0.328	3.03	1.011–9.083	0.048
miR-19b expression	0.455	0.245–0.845	0.013	0.318	0.120–0.846	0.022
Liver cirrhosis	0.897	0.486–1.656	0.728	0.904	0.349–2.339	0.835

*miR-19b* microRNA-19b, *HR* hazard ratio, *CI* confidence interval, *AFP* α-fetoprotein

### Potential targets of miR-19b

In order to evaluate how miR-19b exerts its effect on DFS and recurrence, we knocked down the expression of miR-19b in Hep3B cells, extracted the RNA, and performed an mRNA microarray using the RNA. We then selected genes that were either upregulated or downregulated more that 1.5 times as our candidates. In total, 71 genes were upregulated as miR-19b was knocked down, and 32 genes were downregulated after miR-19b was suppressed (Additional file 1: Table S3 and S4). Among them, genes such as NDRG1 were downregulated when miR-19b was suppressed, whereas EPCAM, HIF1A, HMGB2, and MAPK14 were upregulated. The reported functions of these genes and references are illustrated in Table 3. Then, we tested the expression level of NDRG1, EPCAM, HIF1A, HMGB2 and MAPK 14 in 20 HCC tumor samples from the aforementioned patient cohort, and analyzed the correlation between the expression level of these genes and miR-19b. The results are shown in Additional file 1: Table S5 and Additional files 1 and 2. There was a trend toward negative correlation between the expression of miR-19b and HIF1A and MAPK 14 in our HCC samples (*Pearson’s* correlation, *r* = −0.219 and −0.229, *P* = 0.352 and 0.332, respectively).

**Table 3:** Potential targets of miR-19b

	Expression change after miR-19b knockdown	Functions	Reference
NDRG1	Down regulated	Metastatic suppressor	[38]
EPCAM	Up regulated	Cancer cell stemness	[38–41]
HIF1A	Up regulated	Angiogenesis; Proliferation	[43, 44]
HMGB2	Up regulated	Proliferation; Predict survival of HCC	[45]
MAPK14	Up regulated	Drug resistance	[42]

*NDRG1* N-myc downstream regulated gene 1, *EPCAM* epithelial cell adhesion molecule, *HIF1A* hypoxia inducible factor 1, alpha subunit, *HMGB2* high mobility group box 2, *MAPK14* mitogen-activated protein kinase 14

## Discussion

Currently, surgery remains one of the most effective ways to cure HCC. Traditionally, surgical resection is only recommended for patients with BCLC stage A disease. With the improvements in surgical technique and careful patient selection, however, patients with portal vein invasion or multifocal tumors may also benefit from surgery in terms of DFS [26, 27]. In other words, some patients with more advanced HCC may have a long DFS after surgery, while others experience early recurrence. It is crucial to differentiate between these patients.

In the present study, we first identified miR-19b as our target miRNA using a miRNA microarray of 5 paired samples from HCC patients. MiR-19b was uniformly overexpressed in all samples, indicating its importance in HCC. We also found that let-7 families were downregulated, while miR-21, miR-17, miR-20a, and miR-106b were upregulated in the tumors compared with corresponding non-tumor samples. These findings were consistent with previous reports. We then validated the overexpression of miR-19 using real-time PCR. Interestingly, we found that, the expression of miR-19b was significantly lower in patients who had recurrences within 2 years of curative surgery than in those who remained disease-free at 2 years. This finding suggested that miR-19b might be a prognostic factor for recurrence in this group of patients.

In further analysis, we showed that in patients with HBV-associated HCC presenting with multiple tumors or vascular invasion, a high expression level of miR-19b predicted better DFS after curative surgery compared with that in those with a low expression level. High expression of miR-19b also predicted better DFS and OS in Cox multivariate analysis. Interestingly, one of the variables in multivariate analysis is vascular invasion, as shown in Table 2. We included only patients with HCC at BCLC stage B or C without extrahepatic metastases. The presence of vascular invasion represented stage C disease, whereas absence of vascular invasion was equivalent to stage B. The results of multivariate analysis indicated that the miR-19b expression level correlated with both DFS and OS independent of the BCLC stage. Based on these results, miR-19b may be a useful marker for clinical decision-making in terms of whether or not to perform surgery or the frequency of follow-ups after surgery.

AFP was previously reported to be a prognostic factor for HCC [28]. In the present study, we found that AFP correlated with worse DFS in multivariate analysis. This finding is consistent with previous reports. However, AFP did not predict OS in the present study. As a prognostic factor, miR-19b may be more powerful than AFP. Table 1 shows that more patients in the high-expression miR-19b group had elevated serum AFP. However, when we compared the AFP serum level of patients with high-expression miR-19b with their counterparts, there was no significant difference. In addition, there was no significant correlation between the miR-19b expression level and serum AFP level. Therefore, it was unlikely that there was an association between miR-19b expression and the serum AFP level.

Several reports have demonstrated the oncogenic roles of miR-19b, such as in cancer proliferation, migration, and chemoresistance [21, 29–31]. However, Yin et al. showed that miR-19b inhibited angiogenesis [24]. Zhang et al. revealed that in breast cancer, miR-19b negatively regulated tissue factor, which is important in tumor angiogenesis and metastasis [22]. The role of miR-19b had not been elucidated in HCC. Our microarray results after knockdown of miR-19b offer more support.

Although it is a general concept that genes overexpressed in tumors may be oncogenic, there are exceptions. Huynh demonstrated that retinoblastoma 2 protein (pRb/130), a tumor suppressor gene that is commonly downregulated in cancer, was overexpressed in HCC [32]. In this study, the author showed that pRb/130 was elevated in the majority of HCC samples, but still functioned as a tumor suppressor. Another example is p16^Ink4a^, which is a tumor suppressor but was found to be overexpressed in human papilloma virus (HPV)-related cancer. The overexpression of p16^Ink4a^ was correlated with better treatment response and prognosis [33–37]. Similar to pRb/130 and p16^Ink4a^, we showed that miR-19b was overexpressed in HCC compared with non-tumorous liver tissue, and a higher level of miR-19b was correlated with better survival after curative surgery. Overexpression of miR-19b might be an attempt to stop cell proliferation. MiR-19b might slow down cancer progression, and therefore, its overexpression is correlated with better survival. However, detailed mechanisms will have to be revealed and validated in future studies.

As shown in Table 3, the expression levels of several genes were regulated by miR-19b. NDRG1 is a tumor suppressor that inhibits tumor progression and metastasis, and overexpression of NDRG1 has been shown to exert an anti-metastatic effect [38]. It was downregulated when miR-19b was knocked down. The existence of cancer stem cells was considered to be a cause of tumor recurrence. In HCC, cells expressing EPCAM had the ability to self-renew and to initiate highly invasive HCC, which indicated a worse prognosis [39–41]. Our microarray also revealed that while miR-19b was knocked down, the expression level of EPCAM increased. Similarly, MAPK14 was overexpressed after suppression of miR-19b. MAPK14 has been shown to take part in drug resistance [42]. HIF1A, an important factor also promoting epithelial-to-mesenchymal transition in HCC under hypoxia, was also upregulated as miR-19b was knocked down [43, 44]. HMGB2 is an oncogene promoting proliferation. Kwon et al. revealed that its overexpression indicates a poor HCC prognosis [45]. Through direct or indirect regulation, miR-19b may suppress the function of EPCAM, MAPK14, and HIF1A, as well as HMGB2, and promote the effect of NDRG1, thus suppressing HCC recurrence after curative surgery. In human tumor samples, we also revealed that there was a trend toward negative correlation between the expression of miR-19b and MAPK14 and HIF1A, as shown in Additional file 1: Table S5 and Additional file 2: Figure S1 and Additional file 3: Figure S2. These findings also support that miR-19b targets MAPK14 and HIF1A in vivo.

## Conclusions

In patients with more advanced HBV-associated HCC, the miR-19b expression level in HCC tumor correlated with better DFS and OS after curative surgery. MiR-19b may regulate several genes involved in the metastasis process. MiR-19b may be a novel and useful prognostic factor in patients with more advanced HCC after curative surgery.

## Additional files

Additional file 1: **Supplementary tables. Table S1.** Demographics of selected patients. **Table S2.** The log2 ratio of the expression of selected microRNA from five pairs of tumor and non-tumor liver tissue. The microRNA microarray was performed by SurePrint G3 ChIP/CH3 1X1M array (Agilent Technologies, Santa Clara, CA). **Table S3.** Genes that were overexpressed with ratio over 1.5 times after miR-19b knockdown in Hep3B. mRNA microarray was performed by Human OneArray® (Phalanx Biotech Group, Inc., R.O.C). **Table S4.** Genes that were suppressed with ratio over 1.5 times after miR-19b knockdown in Hep3B. mRNA microarray was performed by Human OneArray® (Phalanx Biotech Group, Inc., R.O.C). **Table S5.** Correlation between the expressions of putative target genes and miR-19b in 20 tumor samples. (DOC 198 kb)
Additional file 2: **Correlation of the expression level between miR-19b and MAPK14.** The expression levels of miR-19b and MAPK14 were determined in 20 resected HCC tumors using real-time PCR. There is a trend toward a negative correlation between miR-19b and MAPK14 (*Pearson’s* correlation, *r* = −0.229, *P* = 0.332). miR-19b, microRNA-19b. HCC, hepatocellular carcinoma. (TIFF 256 kb)
Additional file 3: **Correlation of the expression level between miR-19b and HIF1A.** The expression levels of miR-19b and HIF1A were determined in 20 resected HCC tumors using real-time PCR. There is a trend toward a negative correlation between miR-19b and HIF1A (*Pearson’s* correlation, *r* = −0.219, *P* = 0.352). miR-19b, microRNA-19b. HCC, hepatocellular carcinoma. (TIFF 247 kb)

## Abbreviation

BCLC: Barcelona-Clinic Liver Cancer
HCC: Hepatocellular carcinoma
HBV: Hepatitis B virus
miRNA: microRNA
mRNA: Messenger RNA
qRT-PCR: Quantitative real-time polymerase chain reaction
