# Supplementary material for: Upregulation of MicroRNA-19b predicts good prognosis in patients with hepatocellular carcinoma presenting with vascular invasion or multifocal disease
Source: BMC Cancer. 2015 Oct 9;15:665. doi: 10.1186/s12885-015-1671-5 (PMC4600317; doi:10.1186/s12885-015-1671-5)
Supplement: Additional file 1: — Supplementary tables. Table S1. Demographics of selected patients. Table S2. The log2 ratio of the expression of selected microRNA from five pairs of tumor and non-tumor liver tissue. The microRNA microarray was performed by SurePrint G3 ChIP/CH3 1X1M array (Agilent Technologies, Santa Clara, CA). Table S3. Genes that were overexpressed with ratio over 1.5 times after miR-19b knockdown in Hep3B. mRNA microarray was performed by Human OneArray® (Phalanx Biotech Group, Inc., R.O.C). Table S4. Genes that were suppressed with ratio over 1.5 times after miR-19b knockdown in Hep3B. mRNA microarray was performed by Human OneArray® (Phalanx Biotech Group, Inc., R.O.C). Table S5. Correlation between the expressions of putative target genes and miR-19b in 20 tumor samples. (DOC 198 kb) [file 12885_2015_1671_MOESM1_ESM.doc]

| **Supplementary Table 1** Demographics of selected patients | | | |
| --- | --- | --- | --- |
|  |  |  |  |
|  | Tumor number | |  |
| Vascular invasion | Solitary | Multiple | Total |
| Presence | 48 | 14 | 62 |
| Absence | 10 | 9 | 19 |
| Total | 58 | 23 | 81 |

**Supplementary Table 2.** The log2 ratio of the expression of selected microRNA from five pairs of tumor and non-tumor liver tissue. The microRNA microarray was performed by ­­­SurePrint G3 ChIP/CH3 1X1M array (Agilent Technologies, Santa Clara, CA).

|  | Tumor versus non-tumor ratio in log2 | | | | |
| --- | --- | --- | --- | --- | --- |
|  | S1 | S2 | S3 | S4 | S5 |
| hsa-let-7b | 0.300336157 | 0.038021993 | -2.950415436 | -3.544875378 | -1.381235983 |
| hsa-let-7c | -0.958417314 | -0.931070054 | -4.502725897 | -2.35095123 | -1.946360001 |
| hsa-miR-122 | 1.248844479 | -0.714612654 | -1.394234464 | -0.201131722 | -0.348076374 |
| hsa-miR-17 | 2.990770327 | 0.787407411 | 0.934110317 | 3.894937161 | 3.11012761 |
| hsa-miR-19b | 4.659420945 | 2.056776487 | 1.996860314 | 2.711790816 | 2.957509005 |
| hsa-miR-20a | 2.937356381 | 1.041745693 | 1.085438937 | 3.68872854 | 2.818811789 |
| hsa-miR-21 | 5.166213774 | 3.472309714 | 3.355006618 | 2.734328078 | 4.700675253 |
| hsa-miR-92a | 0.765997619 | -0.731852884 | -0.568294591 | 2.777302467 | 1.197972071 |
| hsa-miR-106b | 3.041004723 | 2.752148549 | 2.50931385 | 1.606156516 | 3.474795259 |

S1=sample 1; S2=sample 2; S3=sample 3; S4=sample 4; S5=sample 5.

**Supplementary Table 3.** Genes that were overexpressed with ratio over 1.5 times after miR-19b knockdown in Hep3B. mRNA microarray was performed by Human OneArray® (Phalanx Biotech Group, Inc., R.O.C)

|  | **Normalized Intensity** | | **log2 (Ratio)** | **P-value** |
| --- | --- | --- | --- | --- |
| **Gene_symbol** | **Ctrl** | **19b** | **19b/Ctrl** | **19b/Ctrl** |
| [A2M](http://www.ncbi.nlm.nih.gov/sites/entrez?db=gene&cmd=search&term=A2M) | 142.711731 | 241.487366 | 0.735915 | 0.014108 |
| ACOT1|ACOT2 | 142.308075 | 244.88295 | 0.742079 | 0.023198 |
| [ARPC2](http://www.ncbi.nlm.nih.gov/sites/entrez?db=gene&cmd=search&term=ARPC2) | 1114.40283 | 1794.53247 | 0.710605 | 0.036271 |
| [ASF1A](http://www.ncbi.nlm.nih.gov/sites/entrez?db=gene&cmd=search&term=ASF1A) | 141.236298 | 273.180359 | 0.915195 | 0.002034 |
| [C14orf126](http://www.ncbi.nlm.nih.gov/sites/entrez?db=gene&cmd=search&term=C14orf126) | 280.247803 | 430.356476 | 0.591303 | 0.023585 |
| [CAPRIN1](http://www.ncbi.nlm.nih.gov/sites/entrez?db=gene&cmd=search&term=CAPRIN1) | 1013.97314 | 1580.76147 | 0.648357 | 0.004748 |
| [CCDC132](http://www.ncbi.nlm.nih.gov/sites/entrez?db=gene&cmd=search&term=CCDC132) | 94.358582 | 150.321457 | 0.651224 | 0.02511 |
| [CCT2](http://www.ncbi.nlm.nih.gov/sites/entrez?db=gene&cmd=search&term=CCT2) | 311.07431 | 488.489197 | 0.646317 | 0.030748 |
| [CD46](http://www.ncbi.nlm.nih.gov/sites/entrez?db=gene&cmd=search&term=CD46) | 84.782135 | 140.886536 | 0.707124 | 0.029807 |
| [CD58](http://www.ncbi.nlm.nih.gov/sites/entrez?db=gene&cmd=search&term=CD58) | 326.872681 | 486.768188 | 0.675717 | 0.002622 |
| [CGRRF1](http://www.ncbi.nlm.nih.gov/sites/entrez?db=gene&cmd=search&term=CGRRF1) | 121.345688 | 192.521027 | 0.627289 | 0.019858 |
| [CLDN12](http://www.ncbi.nlm.nih.gov/sites/entrez?db=gene&cmd=search&term=CLDN12) | 75.400574 | 178.374756 | 1.209784 | 0.001343 |
| [CNOT7](http://www.ncbi.nlm.nih.gov/sites/entrez?db=gene&cmd=search&term=CNOT7) | 595.625488 | 892.791626 | 0.587396 | 0.024955 |
| [CRBN](http://www.ncbi.nlm.nih.gov/sites/entrez?db=gene&cmd=search&term=CRBN) | 280.93219 | 427.136627 | 0.591939 | 0.010284 |
| [DCK](http://www.ncbi.nlm.nih.gov/sites/entrez?db=gene&cmd=search&term=DCK) | 1408.83203 | 2199.17432 | 0.642144 | 0.000457 |
| [DIP2C](http://www.ncbi.nlm.nih.gov/sites/entrez?db=gene&cmd=search&term=DIP2C) | 77.950104 | 106.657227 | 0.924063 | 0.000908 |
| [DKK1](http://www.ncbi.nlm.nih.gov/sites/entrez?db=gene&cmd=search&term=DKK1) | 784.672119 | 1467.479 | 0.901911 | 0.000224 |
| [DNTTIP2](http://www.ncbi.nlm.nih.gov/sites/entrez?db=gene&cmd=search&term=DNTTIP2) | 85.98848 | 131.100113 | 0.710111 | 0.034432 |
| [EIF1](http://www.ncbi.nlm.nih.gov/sites/entrez?db=gene&cmd=search&term=EIF1) | 246.245422 | 373.588318 | 0.592543 | 0.020629 |
| [EIF4H](http://www.ncbi.nlm.nih.gov/sites/entrez?db=gene&cmd=search&term=EIF4H) | 174.649445 | 297.347412 | 0.760231 | 0.021932 |
| [EPCAM](http://www.ncbi.nlm.nih.gov/sites/entrez?db=gene&cmd=search&term=EPCAM) | 1153.65051 | 1735.96753 | 0.589715 | 0.00394 |
| [EPS15](http://www.ncbi.nlm.nih.gov/sites/entrez?db=gene&cmd=search&term=EPS15) | 165.745773 | 295.123535 | 0.804597 | 0.000565 |
| [ERI1](http://www.ncbi.nlm.nih.gov/sites/entrez?db=gene&cmd=search&term=ERI1) | 260.24353 | 420.675018 | 0.685702 | 0.012748 |
| [FAM76B](http://www.ncbi.nlm.nih.gov/sites/entrez?db=gene&cmd=search&term=FAM76B) | 91.945915 | 156.238708 | 0.731157 | 0.010793 |
| [FYTTD1](http://www.ncbi.nlm.nih.gov/sites/entrez?db=gene&cmd=search&term=FYTTD1) | 454.725586 | 682.875244 | 0.588289 | 0.038298 |
| [GART](http://www.ncbi.nlm.nih.gov/sites/entrez?db=gene&cmd=search&term=GART) | 72.458969 | 138.972717 | 0.922535 | 0.001333 |
| [GPN1](http://www.ncbi.nlm.nih.gov/sites/entrez?db=gene&cmd=search&term=GPN1) | 146.732071 | 236.114471 | 0.671147 | 0.047264 |
| [HIF1A](http://www.ncbi.nlm.nih.gov/sites/entrez?db=gene&cmd=search&term=HIF1A) | 231.372711 | 356.004974 | 0.586501 | 0.028085 |
| [HMGB2](http://www.ncbi.nlm.nih.gov/sites/entrez?db=gene&cmd=search&term=HMGB2) | 449.236755 | 686.158569 | 0.613934 | 0.042561 |
| [HNF4G](http://www.ncbi.nlm.nih.gov/sites/entrez?db=gene&cmd=search&term=HNF4G) | 90.674622 | 149.672119 | 0.697593 | 0.014655 |
| [IFNAR2](http://www.ncbi.nlm.nih.gov/sites/entrez?db=gene&cmd=search&term=IFNAR2) | 275.44104 | 459.288574 | 0.722952 | 0.005628 |
| [INTS12](http://www.ncbi.nlm.nih.gov/sites/entrez?db=gene&cmd=search&term=INTS12) | 146.862 | 226.813812 | 0.592573 | 0.031335 |
| [KLHDC2](http://www.ncbi.nlm.nih.gov/sites/entrez?db=gene&cmd=search&term=KLHDC2) | 623. | 956.368042 | 0.616829 | 0.001628 |
| [LAP3](http://www.ncbi.nlm.nih.gov/sites/entrez?db=gene&cmd=search&term=LAP3) | 222.935318 | 343.967834 | 0.594912 | 0.031122 |
| [LMAN1](http://www.ncbi.nlm.nih.gov/sites/entrez?db=gene&cmd=search&term=LMAN1) | 481.341553 | 748.282471 | 0.619503 | 0.015717 |
| [LYPLA1](http://www.ncbi.nlm.nih.gov/sites/entrez?db=gene&cmd=search&term=LYPLA1) | 423.843445 | 722.704468 | 0.761751 | 0.001643 |
| [LYZ](http://www.ncbi.nlm.nih.gov/sites/entrez?db=gene&cmd=search&term=LYZ) | 335.052551 | 629.326904 | 0.911164 | 0.003048 |
| [MAD2L1](http://www.ncbi.nlm.nih.gov/sites/entrez?db=gene&cmd=search&term=MAD2L1) | 462.634033 | 830.118408 | 0.851077 | 0.008231 |
| [MAPK14](http://www.ncbi.nlm.nih.gov/sites/entrez?db=gene&cmd=search&term=MAPK14) | 137.092987 | 250.687927 | 0.86836 | 0.047774 |
| [MCOLN1](http://www.ncbi.nlm.nih.gov/sites/entrez?db=gene&cmd=search&term=MCOLN1) | 333.577118 | 514.047668 | 0.634371 | 0.022188 |
| [MIR17HG](http://www.ncbi.nlm.nih.gov/sites/entrez?db=gene&cmd=search&term=MIR17HG) | 434.429016 | 763.419861 | 0.812348 | 0.00192 |
| [NUP35](http://www.ncbi.nlm.nih.gov/sites/entrez?db=gene&cmd=search&term=NUP35) | 225.489502 | 398.93689 | 0.814236 | 0.011764 |
| [NUP35](http://www.ncbi.nlm.nih.gov/sites/entrez?db=gene&cmd=search&term=NUP35) | 156.238922 | 327.092407 | 1.046843 | 0.000201 |
| [NUP54](http://www.ncbi.nlm.nih.gov/sites/entrez?db=gene&cmd=search&term=NUP54) | 327.002563 | 546.565796 | 0.724584 | 0.001042 |
| [ODC1](http://www.ncbi.nlm.nih.gov/sites/entrez?db=gene&cmd=search&term=ODC1) | 157.442932 | 255.340698 | 0.66681 | 0.014234 |
| [PDCD4](http://www.ncbi.nlm.nih.gov/sites/entrez?db=gene&cmd=search&term=PDCD4) | 381.988251 | 672.932617 | 0.812454 | 0.000433 |
| [PET117](http://www.ncbi.nlm.nih.gov/sites/entrez?db=gene&cmd=search&term=PET117) | 126.363571 | 185.917801 | 0.851311 | 0.00121 |
| [PLDN](http://www.ncbi.nlm.nih.gov/sites/entrez?db=gene&cmd=search&term=PLDN) | 247.447113 | 377.562439 | 0.594265 | 0.006214 |
| [PRDX3](http://www.ncbi.nlm.nih.gov/sites/entrez?db=gene&cmd=search&term=PRDX3) | 105.473106 | 189.962738 | 0.999121 | 0.011848 |
| [PSMC2](http://www.ncbi.nlm.nih.gov/sites/entrez?db=gene&cmd=search&term=PSMC2) | 1361.47644 | 2067.53955 | 0.614495 | 0.009363 |
| [RCN2](http://www.ncbi.nlm.nih.gov/sites/entrez?db=gene&cmd=search&term=RCN2) | 291.580414 | 459.891541 | 0.643419 | 0.014188 |
| [RCVRN](http://www.ncbi.nlm.nih.gov/sites/entrez?db=gene&cmd=search&term=RCVRN) | 330.287537 | 523.272644 | 0.656096 | 0.004233 |
| [RDX](http://www.ncbi.nlm.nih.gov/sites/entrez?db=gene&cmd=search&term=RDX) | 3091.05908 | 2498.55981 | 0.689268 | 0.013346 |
| [RNF11](http://www.ncbi.nlm.nih.gov/sites/entrez?db=gene&cmd=search&term=RNF11) | 131.66449 | 225.063538 | 0.75696 | 0.036616 |
| RPE|LOC729020 | 113.042862 | 182.378174 | 0.674451 | 0.031277 |
| [SERPINI1](http://www.ncbi.nlm.nih.gov/sites/entrez?db=gene&cmd=search&term=SERPINI1) | 97.708481 | 144.826508 | 0.740212 | 0.048231 |
| [SF3B1](http://www.ncbi.nlm.nih.gov/sites/entrez?db=gene&cmd=search&term=SF3B1) | 133.135284 | 224.209137 | 0.728584 | 0.017016 |
| [SLC25A32](http://www.ncbi.nlm.nih.gov/sites/entrez?db=gene&cmd=search&term=SLC25A32) | 127.572235 | 207.148193 | 0.662014 | 0.007683 |
| [SNORD36C](http://www.ncbi.nlm.nih.gov/sites/entrez?db=gene&cmd=search&term=SNORD36C) | 418.01593 | 714.834351 | 0.768095 | 0.000763 |
| [TBCE](http://www.ncbi.nlm.nih.gov/sites/entrez?db=gene&cmd=search&term=TBCE) | 163.13591 | 269.528442 | 0.699083 | 0.009059 |
| [TIMM23](http://www.ncbi.nlm.nih.gov/sites/entrez?db=gene&cmd=search&term=TIMM23) | 697.56311 | 1115.50928 | 0.699465 | 0.016916 |
| [TMEM14B](http://www.ncbi.nlm.nih.gov/sites/entrez?db=gene&cmd=search&term=TMEM14B) | 113.110138 | 249.291611 | 1.641563 | 0.00001 |
| [TMPO](http://www.ncbi.nlm.nih.gov/sites/entrez?db=gene&cmd=search&term=TMPO) | 87.795654 | 161.963135 | 0.86422 | 0.003373 |
| [TSEN15](http://www.ncbi.nlm.nih.gov/sites/entrez?db=gene&cmd=search&term=TSEN15) | 118.065384 | 191.063675 | 0.677459 | 0.031859 |
| TTC4|HEATR8-TTC4 | 66.09787 | 116.443657 | 1.165988 | 0.000699 |
| [UBE2E1](http://www.ncbi.nlm.nih.gov/sites/entrez?db=gene&cmd=search&term=UBE2E1) | 983.404175 | 788.763489 | 0.669209 | 0.039925 |
| [UBTD2](http://www.ncbi.nlm.nih.gov/sites/entrez?db=gene&cmd=search&term=UBTD2) | 126.099106 | 197.361755 | 0.608803 | 0.004594 |
| [UCHL5](http://www.ncbi.nlm.nih.gov/sites/entrez?db=gene&cmd=search&term=UCHL5) | 2127.70557 | 3183.91284 | 0.597296 | 0.005084 |
| [VPS26A](http://www.ncbi.nlm.nih.gov/sites/entrez?db=gene&cmd=search&term=VPS26A) | 202.513458 | 313.532013 | 0.609134 | 0.026757 |
| [WDR5B](http://www.ncbi.nlm.nih.gov/sites/entrez?db=gene&cmd=search&term=WDR5B) | 76.878326 | 122.72464 | 0.651776 | 0.048347 |
| [ZNF222](http://www.ncbi.nlm.nih.gov/sites/entrez?db=gene&cmd=search&term=ZNF222) | 129.513962 | 200.820816 | 0.593327 | 0.004745 |

**Supplementary Table 4**. Genes that were suppressed with ratio over 1.5 times after miR-19b knockdown in Hep3B. mRNA microarray was performed by Human OneArray® (Phalanx Biotech Group, Inc., R.O.C)

|  | **Normalized Intensity** | | **log2 (Ratio)** | **P-value** |
| --- | --- | --- | --- | --- |
| **Gene_symbol** | **Ctrl** | **19b** | **19b/Ctrl** | **19b/Ctrl** |
| [BCL2L11](http://www.ncbi.nlm.nih.gov/sites/entrez?db=gene&cmd=search&term=BCL2L11) | 346.28537 | 125.592957 | -1.412705 | 0.01326 |
| [BEST1](http://www.ncbi.nlm.nih.gov/sites/entrez?db=gene&cmd=search&term=BEST1) | 150.873047 | 99.675629 | -0.637339 | 0.038466 |
| [CAMK2N2](http://www.ncbi.nlm.nih.gov/sites/entrez?db=gene&cmd=search&term=CAMK2N2) | 268.599731 | 181.213776 | -0.600835 | 0.017554 |
| [CRIP2](http://www.ncbi.nlm.nih.gov/sites/entrez?db=gene&cmd=search&term=CRIP2) | 168.886871 | 104.352806 | -0.739732 | 0.039993 |
| [DOCK10](http://www.ncbi.nlm.nih.gov/sites/entrez?db=gene&cmd=search&term=DOCK10) | 183.683044 | 113.846306 | -0.726712 | 0.033509 |
| [GNAI2](http://www.ncbi.nlm.nih.gov/sites/entrez?db=gene&cmd=search&term=GNAI2) | 103.728561 | 100.061325 | -0.60157 | 0.047913 |
| [HBQ1](http://www.ncbi.nlm.nih.gov/sites/entrez?db=gene&cmd=search&term=HBQ1) | 113.369965 | 78.611244 | -0.73116 | 0.006856 |
| [HMHA1](http://www.ncbi.nlm.nih.gov/sites/entrez?db=gene&cmd=search&term=HMHA1) | 1099.12646 | 719.775146 | -0.659267 | 0.03189 |
| HSPA6|HSPA7 | 3175.1499 | 1757.38354 | -0.833549 | 0.000236 |
| HSPA6|HSPA7 | 2865.51172 | 1474.41675 | -0.950015 | 0.000032 |
| [IFFO1](http://www.ncbi.nlm.nih.gov/sites/entrez?db=gene&cmd=search&term=IFFO1) | 290.835724 | 201.130829 | -0.598061 | 0.00653 |
| [INSR](http://www.ncbi.nlm.nih.gov/sites/entrez?db=gene&cmd=search&term=INSR) | 324.654877 | 218.040436 | -0.599201 | 0.003926 |
| [KIAA0415](http://www.ncbi.nlm.nih.gov/sites/entrez?db=gene&cmd=search&term=KIAA0415) | 2286.8999 | 1300.50928 | -0.809712 | 0.002345 |
| [KIAA0513](http://www.ncbi.nlm.nih.gov/sites/entrez?db=gene&cmd=search&term=KIAA0513) | 198.620712 | 132.879684 | -0.622487 | 0.009206 |
| [LOC100507605](http://www.ncbi.nlm.nih.gov/sites/entrez?db=gene&cmd=search&term=LOC100507605) | 76.268204 | 59.419193 | -0.718616 | 0.035305 |
| [LOC100509196](http://www.ncbi.nlm.nih.gov/sites/entrez?db=gene&cmd=search&term=LOC100509196) | 168.550507 | 100.173615 | -0.786546 | 0.010189 |
| [NDRG1](http://www.ncbi.nlm.nih.gov/sites/entrez?db=gene&cmd=search&term=NDRG1) | 5008.92725 | 3245.94409 | -0.609766 | 0.000943 |
| [OSTalpha](http://www.ncbi.nlm.nih.gov/sites/entrez?db=gene&cmd=search&term=OSTalpha) | 67.835457 | 26.051586 | -1.278903 | 0.015371 |
| [PPP1R10](http://www.ncbi.nlm.nih.gov/sites/entrez?db=gene&cmd=search&term=PPP1R10) | 1256.76892 | 789.60083 | -0.67226 | 0.001866 |
| [PROM1](http://www.ncbi.nlm.nih.gov/sites/entrez?db=gene&cmd=search&term=PROM1) | 230.769531 | 127.787521 | -0.870868 | 0.026028 |
| [RHEBL1](http://www.ncbi.nlm.nih.gov/sites/entrez?db=gene&cmd=search&term=RHEBL1) | 180.337784 | 120.471497 | -0.623636 | 0.015243 |
| [RPS20](http://www.ncbi.nlm.nih.gov/sites/entrez?db=gene&cmd=search&term=RPS20) | 173.712219 | 97.522568 | -0.861215 | 0.022132 |
| [RPS23](http://www.ncbi.nlm.nih.gov/sites/entrez?db=gene&cmd=search&term=RPS23) | 148.534622 | 93.272583 | -0.692757 | 0.021838 |
| [SARDH](http://www.ncbi.nlm.nih.gov/sites/entrez?db=gene&cmd=search&term=SARDH) | 155.227448 | 103.117599 | -0.631133 | 0.016182 |
| [SF3B2](http://www.ncbi.nlm.nih.gov/sites/entrez?db=gene&cmd=search&term=SF3B2) | 2729.12842 | 1786.38879 | -0.601105 | 0.024094 |
| [SMC1A](http://www.ncbi.nlm.nih.gov/sites/entrez?db=gene&cmd=search&term=SMC1A) | 460.857056 | 306.513794 | -0.605314 | 0.009017 |
| [SPTY2D1](http://www.ncbi.nlm.nih.gov/sites/entrez?db=gene&cmd=search&term=SPTY2D1) | 247.644318 | 168.49556 | -0.586263 | 0.04158 |
| [SYMPK](http://www.ncbi.nlm.nih.gov/sites/entrez?db=gene&cmd=search&term=SYMPK) | 107.683945 | 74.637108 | -0.614529 | 0.041359 |
| [TEP1](http://www.ncbi.nlm.nih.gov/sites/entrez?db=gene&cmd=search&term=TEP1) | 327.661438 | 216.185196 | -0.625878 | 0.014573 |
| [UNC13A](http://www.ncbi.nlm.nih.gov/sites/entrez?db=gene&cmd=search&term=UNC13A) | 612.813477 | 405.884277 | -0.61067 | 0.005785 |
| [ZFHX3](http://www.ncbi.nlm.nih.gov/sites/entrez?db=gene&cmd=search&term=ZFHX3) | 115.251373 | 65.71727 | -0.825679 | 0.018797 |
| [ZNF185](http://www.ncbi.nlm.nih.gov/sites/entrez?db=gene&cmd=search&term=ZNF185) | 306.499512 | 204.472717 | -0.612795 | 0.033672 |
| [ZNF611](http://www.ncbi.nlm.nih.gov/sites/entrez?db=gene&cmd=search&term=ZNF611) | 202.17244 | 130.907257 | -0.666698 | 0.002219 |

| **Supplementary Table 5** Correlation between the expressions of putative target genes and miR-19b in 20 tumor samples. | | |
| --- | --- | --- |
|  |  |  |
|  | Pearson's correlation analysis | |
|  | r value | p value |
| NDRG1 | -0.253 | 0.282 |
| EPCAM | 0.232 | 0.325 |
| HIF1A | -0.219 | 0.352 |
| HMGB2 | 0.064 | 0.788 |
| MAPK14 | -0.229 | 0.332 |
