# Supplementary figures and images for: Upregulation of MicroRNA-19b predicts good prognosis in patients with hepatocellular carcinoma presenting with vascular invasion or multifocal disease
Source: BMC Cancer. 2015 Oct 9;15:665. doi: 10.1186/s12885-015-1671-5 (PMC4600317; doi:10.1186/s12885-015-1671-5)

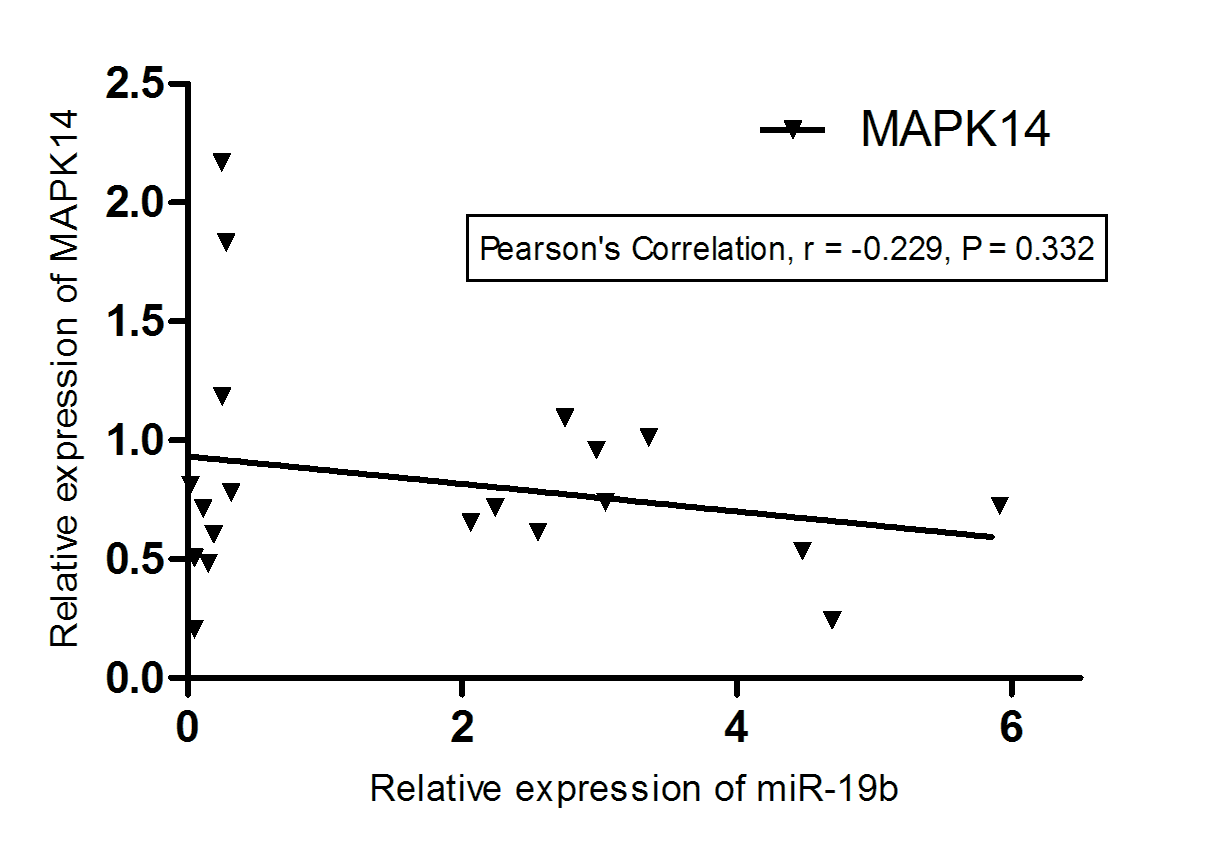

Supplement: Additional file 2: — Correlation of the expression level between miR-19b and MAPK14. The expression levels of miR-19b and MAPK14 were determined in 20 resected HCC tumors using real-time PCR. There is a trend toward a negative correlation between miR-19b and MAPK14 (Pearson’s correlation, r = −0.229, P = 0.332). miR-19b, microRNA-19b. HCC, hepatocellular carcinoma. (TIFF 256 kb) [file 12885_2015_1671_MOESM2_ESM.tiff]

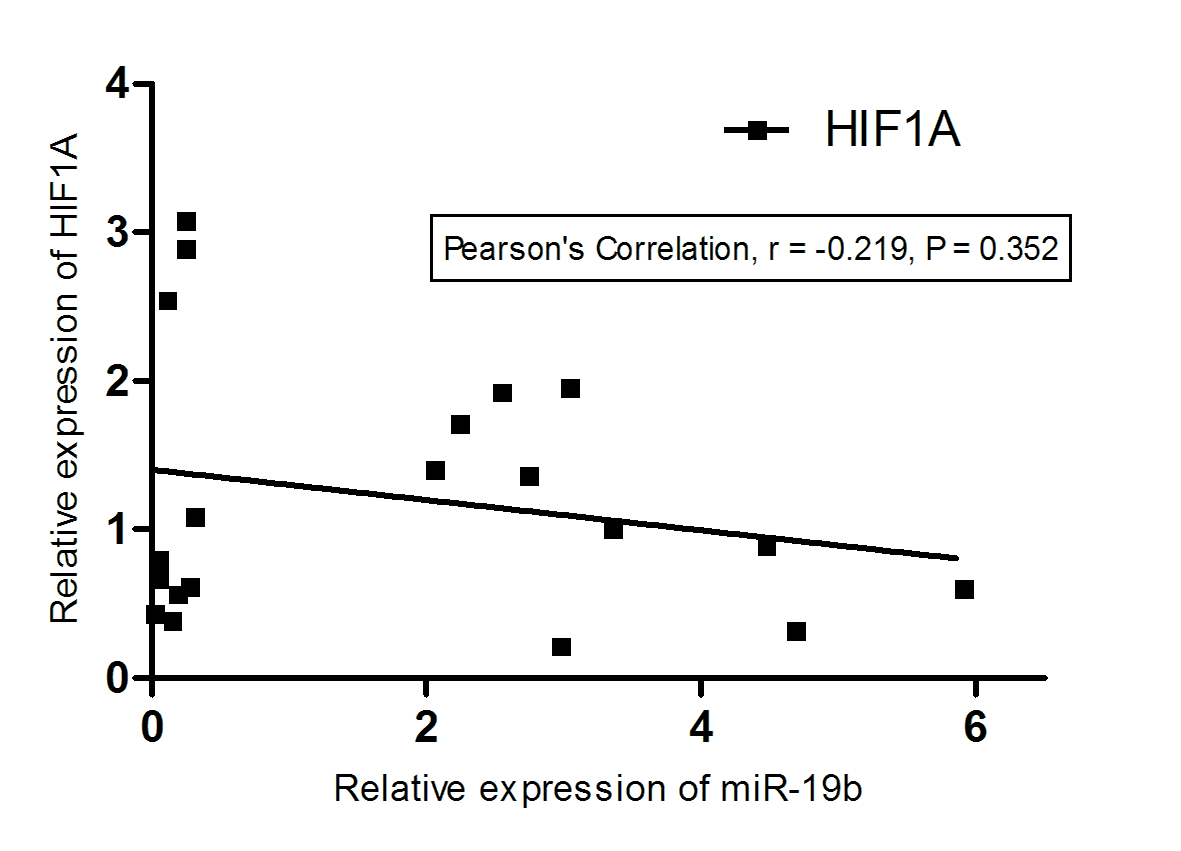

Supplement: Additional file 3: — Correlation of the expression level between miR-19b and HIF1A. The expression levels of miR-19b and HIF1A were determined in 20 resected HCC tumors using real-time PCR. There is a trend toward a negative correlation between miR-19b and HIF1A (Pearson’s correlation, r = −0.219, P = 0.352). miR-19b, microRNA-19b. HCC, hepatocellular carcinoma. (TIFF 247 kb) [file 12885_2015_1671_MOESM3_ESM.tiff]
